# Investigation of the human pathogen *Acinetobacter baumannii *under iron limiting conditions

**DOI:** 10.1186/1471-2164-12-126

**Published:** 2011-02-23

**Authors:** Bart A Eijkelkamp, Karl A Hassan, Ian T Paulsen, Melissa H Brown

**Affiliations:** 1 School of Biological Sciences, Flinders University, Adelaide, SA 5001, Australia; 2 Department of Chemistry and Biomolecular Sciences, Macquarie University, Sydney, NSW 2109, Australia

## Abstract

**Background:**

Iron acquisition systems are important virulence factors in pathogenic bacteria. To identify these systems in *Acinetobacter baumannii*, the transcriptomic response of the completely sequenced strain ATCC 17978 under iron limiting conditions was investigated using a genomic microarray that contained probes for all annotated open reading frames.

**Results:**

Under low iron conditions, transcription levels were more than 2-fold up-regulated for 463 genes, including 95 genes that were up-regulated more than 4-fold. Of particular significance, three siderophore biosynthesis gene clusters, including one novel cluster, were highly up-regulated. Binding sites for the ferric uptake regulator were identified in the promoter regions of many up-regulated genes, suggesting a prominent role for this regulator in the *Acinetobacter *iron acquisition response. Down-regulation under iron limitation was less dramatic as the transcription of only 202 genes varied more than 2-fold. Various genes involved in motility featured prominently amongst the genes down-regulated when iron was less readily available. Motility assays confirmed that these transcriptional changes are manifested at the phenotypic level. The siderophore biosynthesis gene clusters were further investigated by means of comparative genomic analysis of 10 sequenced *Acinetobacter *isolates. These analyses revealed important roles for mobile genetic elements in shaping the siderophore meditated iron acquisition mechanisms between different *Acinetobacter *strains.

**Conclusions:**

*A. baumannii *grown under iron limited conditions resulted in major transcriptional changes of not only many iron acquisition related genes, but also genes involved in other processes such as motility. Overall, this study showed that *A. baumannii *is well adaptable to growth in an environment which has limiting iron availability.

## Background

An increasing prevalence of infections caused by *Acinetobacter baumannii *has been observed in the clinical setting throughout the last 10 to 15 years [1,2]. *A. baumannii *is able to persist in the hospital environment and in particular intensive care units, due to its wide variety of resistance mechanisms and high survival rate on abiotic surfaces [3-6]. Some clinical *A. baumannii *strains have been shown to be naturally competent for the uptake of genetic material, which facilitates acquisition of novel resistance and virulence genes [7-9].

Free iron is a limited micronutrient in hosts where it is typically tightly bound within a range of biomolecules, such as heme. As such, iron acquisition systems are important factors for the virulence of pathogenic organisms. Bacteria can adapt to iron limited host environments through the expression of a range of iron acquisition mechanisms. One pathway for uptake of iron involves direct binding of Fe^2+ ^or heme to receptors or transport proteins on the cell surface [10]. A second more energy intensive mechanism of iron uptake involves the production and secretion of high-affinity iron chelating siderophores, which compete with host cells for iron [11,12]. The genes involved in the production of a siderophore are usually clustered within the genome of the producing organism. In addition to biosysnthesis genes, many of these gene clusters also encode efflux pumps with putative roles in siderophore export. Transporters classified within the ATP-binding cassette (ABC) superfamily, major facilitator superfamily (MFS) and resistance-nodulation-cell division (RND) family have been associated with siderophore extrusion [13]. However, the ability to transport siderophores into the extracellular space has been shown for only two pumps, both MFS members. EntS of *Escherichia coli *transports enterobactin [14] and YmfE of *Bacillus subtilis *is involved in transport of bacillibactin [15]. Inactivation of these pumps results in decreased efflux of the fully synthesized siderophore, but increased extrusion of siderophore precursor products [14,15].

The uptake and reduction of iron-loaded ferric siderophores involves the TonB-ExbB-ExbD energy transduction system in combination with a ferric siderophore complex receptor [16,17]. Bacteria often contain numerous ferric siderophore complex receptors. Some of these are encoded within siderophore biosynthesis gene clusters and are likely to display specificity for the locally encoded siderophore. However, various other ferric siderophore receptors can be scattered throughout the genome and may have the ability to recognize exogneously produced siderophores that are structurally unrelated to endogenous siderophores [18,19].

Most *A. baumannii *strains have the ability to grow under iron limiting conditions, which assists in the colonization of a host, however, a diversity of iron acquisition mechanisms has been shown between different *Acinetobacter *strains [20]. To date, three different siderophore biosynthesis gene clusters have been described in *A. baumannii *[21-23]. Of these, the cluster encoding the siderophore acinetobactin has been the most extensively studied. Knockout experiments have confirmed the functions of both a siderophore biosynthesis protein and receptor from the acinetobactin gene cluster [24]. Furthermore, acinetobactin is the only siderophore produced by *Acinetobacter *to have been structurally characterized [25]. A second siderophore biosynthesis gene cluster found only in *A. baumannii *8399 has been characterized using complementation experiments in an *E. coli *mutant strain [21]. Finally, a putative siderophore biosynthesis gene cluster has been described in strain ATCC 17978 and subjected to limited quantitative reverse transcription PCR (qRT-PCR) analyses under iron limiting conditions [23].

To comprehensively identify mechanisms of iron acquisition and low iron adaptation in *A. baumannii*, the affect of iron starvation on *A. baumannii *cells was investigated at the global level. The transcriptomic responses of *A. baumannii *ATCC 17978 cells to low iron conditions were examined using a whole genome microarray.

## Results and Discussion

### Optimization of test conditions for transcriptomics

**Figure 1:**
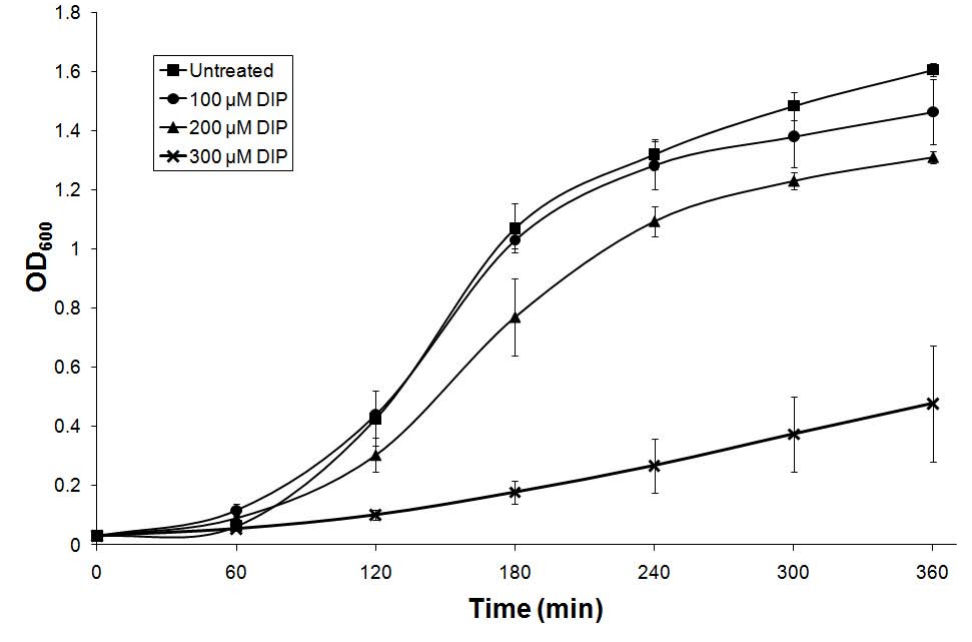
**Growth curves of *A. baumannii *with varying iron concentrations**. Growth under different iron concentrations was tested in Mueller-Hinton (MH) broth and MH supplemented with 2,2'-dipyridyl (DIP) to final concentrations of 100, 200 and 300 μM. Absorbance was measured every hour at OD_600 _for 6 hours; the data represent the average of three separate experiments and the error bars show the standard deviation.

Iron is an essential micronutrient and depletion in the growth medium is likely to have an impact on cell viability. Therefore, growth of *A. baumannii *ATCC 17978 was investigated under varying iron concentrations to determine optimal conditions for whole transcriptome analysis. Reduction of available iron in Mueller-Hinton (MH) medium was achieved by supplementation of 2,2'-dipyridyl (DIP), a synthetic iron chelator. This compound had no effect on the pH of the medium (data not shown). No significant change in the growth rate of *A. baumannii *strain ATCC 17978 was observed after addition of 100 μM DIP, whereas, supplementation with 200 μM DIP resulted in a growth delay of approximately 45 min at mid-log phase (OD_600 _= 0.7) (Figure 1). Moreover, the total biomass was reduced by more than 10% at stationary phase (> 240 min). Addition of 300 μM DIP had a major impact on growth and resulted in more than 70% biomass reduction compared to cultures under iron replete conditions during stationary phase. Due to the moderate inhibitory, but non-lethal effect of 200 μM DIP, this concentration was chosen to study transcriptional changes under iron limitation. Preliminary qRT-PCR was performed to confirm transcriptional adaptation in response to iron limitation by assaying the level of transcription of the ferric uptake regulator (FUR), which is known for auto-up-regulation when iron is limited [26]. *A. baumannii *grown in the presence of 200 μM DIP expressed *FUR *at levels increased more than 2-fold compared to cells grown under iron replete conditions (data not shown). Therefore, the genome wide transcriptional changes of ATCC 17978 grown in MH medium and MH supplemented with 200 μM DIP during mid log-phase were compared by microarray analysis.

### Global transcriptional changes of *A. baumannii *to iron starvation

**Figure 2:**
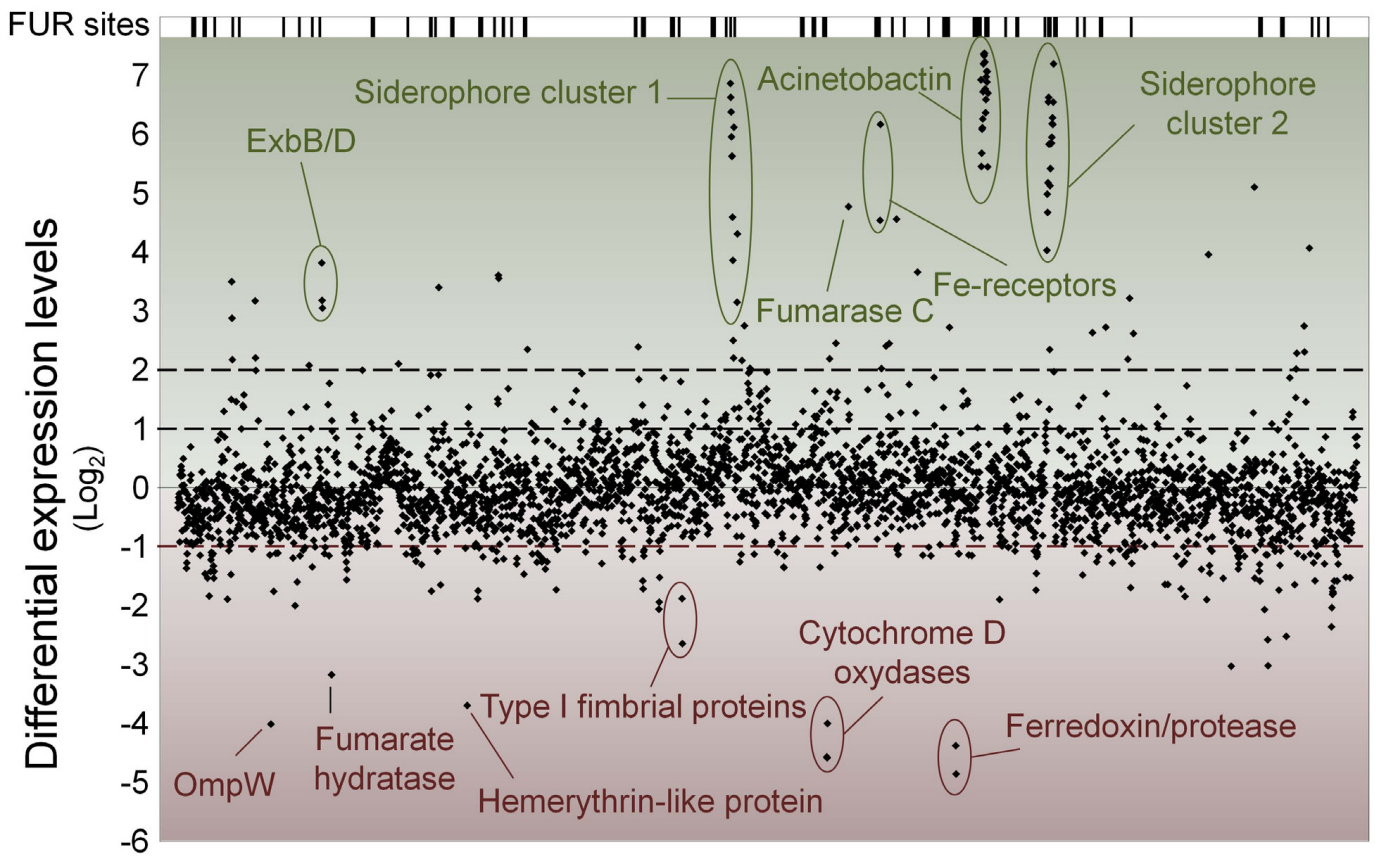
**Overview of transcriptional responses to iron starvation**. The *A. baumannii *ATCC 17978 transcriptome was compared under iron replete and iron limiting conditions (200 μM DIP). All 3367 genes of the *A. baumannii *ATCC 17978 genome are represented on the X-axis, ordered according locus tag. Differential expression levels between iron replete and iron limiting conditions are displayed in Log_2_-values on the Y-axis. Up- and down-regulated genes under iron limiting conditions are displayed in the green and red sections, respectively. Gene names or functions have been provided for various highly differentially expressed genes, such as siderophore biosynthesis genes and the fumarases. The putative FUR binding sites displayed in the top section were identified as described in Methods and the nucleotide sequences are listed in Additional file 2.

**Figure 3:**
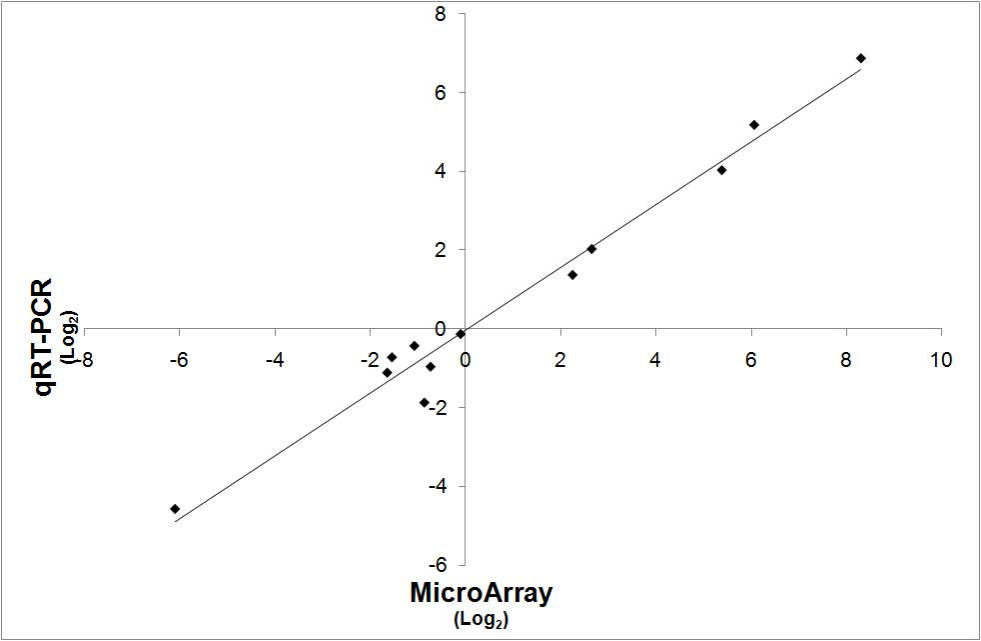
**Validation of the microarray results**. The transcriptomic results obtained by microarray hybridisation were validated by quantitive RT-PCR (qRT-PCR) analysis. The level of differential expression of 12 genes was compared and showed a correlation between microarray (Y-axis) and qRT-PCR analysis (X-axis). The level of differential expression between iron replete and iron limitation is given in Log_2_-values.

Iron limitation had far reaching transcriptional effects on *A. baumannii *cells (Figure 2). Significance analysis [27] of the microarray data showed that 1207 genes were significantly differentially expressed under iron limiting as compared to iron replete conditions (Additional file 1). Transcript levels were more than 2-fold higher for 463 genes, of which 95 genes were up-regulated more than 4-fold (Figure 2). The maximum overexpression observed was 165-fold for the siderophore biosynthesis gene *basD*. Fewer genes were down-regulated under iron limitation; only 202 genes were more than 2-fold underexpressed with a maximum down-regulation of 29-fold (A1S_2297). The array results were validated by qRT-PCR analysis on a subset of differentially expressed genes (Figure 3). There was good correlation between data from the qRT-PCR and the microarray analyses, although the qRT-PCR data generally showed higher fold changes than the microarray expression data. The tendency for microarrays to underestimate fold changes relative to qRT-PCR is well established [28].

**Figure 4:**
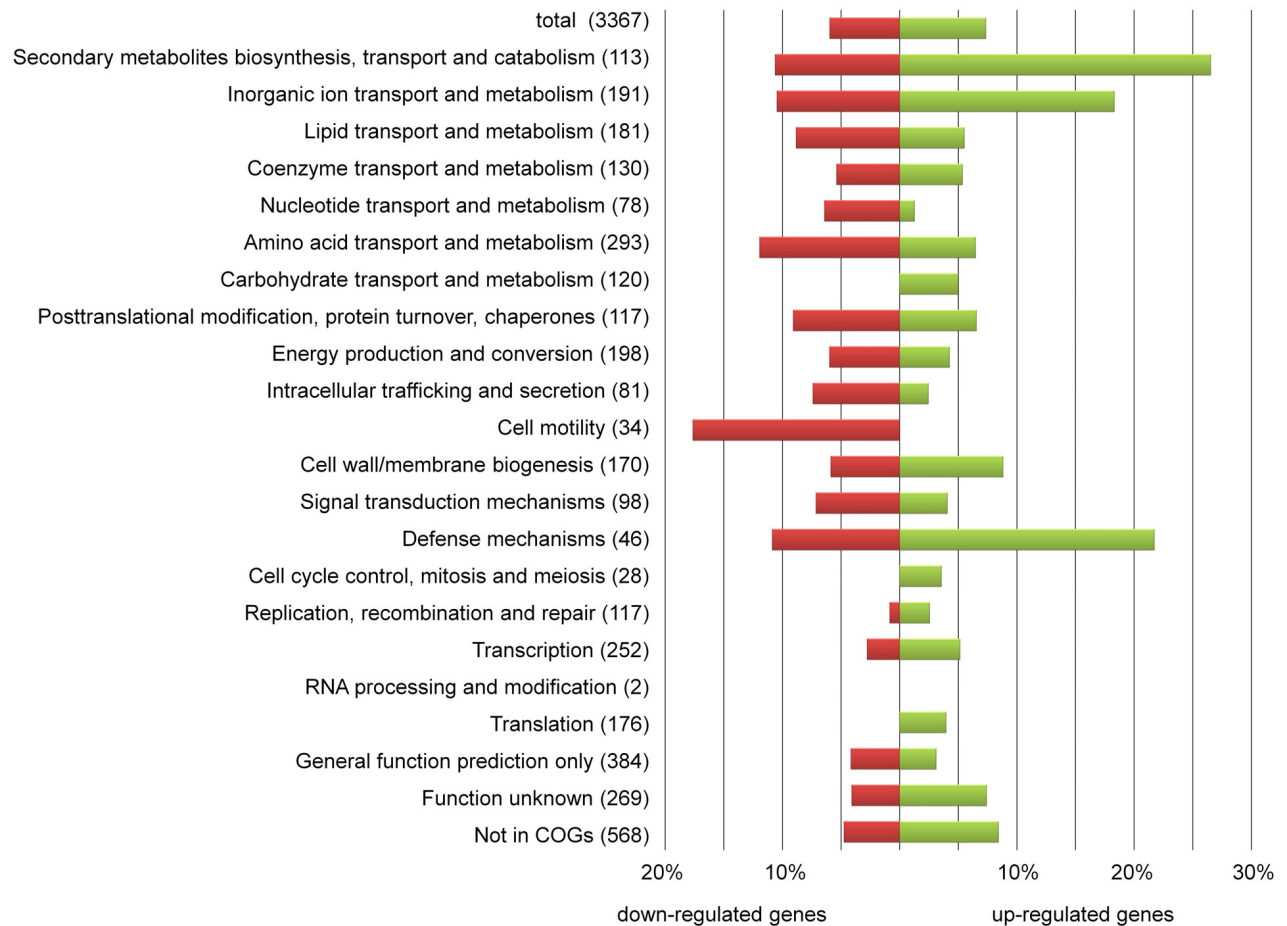
**Microarray results displayed by COG-function**. Depiction of cluster of orthologous groups (COG) and the percentage of up-regulated (green) and down-regulated genes (red) within such group determined by microarray. The total number of genes per COG is shown in parentheses.

Microarray data displayed by clusters of orthologous groups (COG) functional categories showed that 27% of the genes up-regulated under iron limited conditions encode proteins involved in secondary metabolite biosynthesis, transport and catabolism (Figure 4). The majority of these genes are located within three large overexpressed gene clusters, each of which is known or predicted to synthesize a siderophore (Figure 2). Siderophore cluster 1 (A1S_1647-1657) is a novel putative siderophore gene cluster, having not been previously identified. The two other highly overexpressed siderophore clusters identified, siderophore cluster 2 (A1S_2562-2581) and the acinetobactin cluster (A1S_2372-2392), have been described previously [21,23]. Many other overexpressed genes within this COG category encode ferric siderophore receptors, which are widely dispersed across the genome.

In addition to siderophore-related genes, a high percentage, 22%, of genes up-regulated under iron limitation, encoded proteins categorized within the defence mechanism COG. A number of these genes encode transporter proteins that are classified as defence proteins due to their predicted roles in the export of metabolic waste or other toxic compounds. Interestingly, our analysis of several of these proteins, primarily those encoded within siderophore biosynthetic loci, suggested that they function in the extrusion of siderophores (Hassan *et al.*, unpublished data).

Various genes related to cell motility were down-regulated when *A. baumannii *was grown under iron limiting conditions (Figure 4). These included biosynthesis genes homologous to both type IV pili and chaperone-usher pili assembly systems, or type I pili. Another heavily down-regulated gene encoded a hemerythrin-like protein (A1S_0891). Various functions have been suggested for these proteins with an iron containing centre, including detoxification, transport and storage of iron and/or oxygen, or a role as a sensory protein [29]. The cytochrome D genes, part of the respiratory system, and 4Fe-4S-ferredoxin, which facilitates electron transport in various metabolic processes, were also down-regulated more than 4-fold. The iron-dependent Class I fumarate hydratase was found to be 9-fold down-regulated. In contrast, fumarase C, which belongs to the iron-independent Class II was 27-fold up-regulated, suggesting a physiological shift to the Class II protein under iron limitation (Figure 2). These findings correlated with results observed in a study on fumarase A (Class I) and fumarase C in the tri-carboxylic acid cycle of *Pseudomonas fluorescence *[30]. In conclusion, *A. baumannii *showed strong transcriptional responses to iron starvation, predominantly in up-regulation of iron acquisition mechanisms. However, many genes related to other processes than iron acquisition, such as respiration and motility, were also transcriptionally affected under the conditions tested.

### *A. baumannii *FUR box optimization

**Figure 5:**
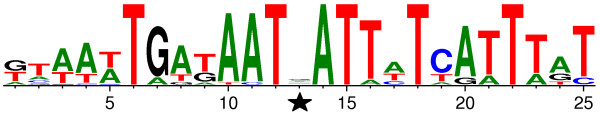
**The optimized *A. baumannii *FUR motif**. Per position, the size of the nucleotide (T in red, A in green, C in blue and G in black) indicates its prevalence in the 22 included sequences from Table 1. The motif shows a palindrome with a central non-conserved nucleotide in position 13 which is indicated by the star. The figure of the *A. baumannii *ATCC 17978 FUR motif was created using WebLogo 3.0 [64].

Bioinformatic analyses were performed to identify motifs within the promoter regions of iron responsive genes that could serve regulatory functions. The multiple em for motif elicitation (MEME) tool and the multiple alignment and search tool (MAST) were used to identify motifs and search for these motifs across the *A. baumannii *ATCC 17978 genome, respectively [31,32]. MEME-based analyses of the upstream regions of all genes overexpressed by at least 4-fold under iron limitation identified a motif bearing strong similarity to the FUR binding sites of *E. coli *and *Pseudomonas *spp. [33]. To confirm the importance of FUR in regulating iron adaptation responses in *A. baumannii*, a scoring matrix was created using the experimentally determined *E. coli *FUR binding sites [34]. This scoring matrix was used to screen the ATCC 17978 genome using MAST. Hits obtained using MAST, which were found upstream of genes that were more than 4-fold up-regulated in response to iron limitation and with a p-value less than 10e^-5^, were selected for iterative refinement of the scoring matrix, until no new hits were obtained (Table 1). The resulting *A. baumannii *FUR box motif showed a 25 nucleotide palindromic sequence (Figure 5).

**Table 1:** Putative FUR binding sequences in the ATCC 17978 genome

Locus tag	Putative FUR binding sequence (5'-3')
A1S_0242	TTATTTGGTAATTATTCTCATTTAT
A1S_0416	GGATTTGTTAATGATTATCATTTGC
A1S_0474	GCGAATAATAATAATTCTTATTTAT
A1S_0980	GATATTGTTAATAATTATCATTATT
A1S_1647	TGAAATGATAATAATTATCATTAAT
A1S_1657	ATAATTGATAATGATAATCATTTTT
A1S_1667	GATAATGTAAATAATTCTCATTTAT
A1S_2077	TCATTTGATACTGATTATCAATATT
A1S_2080	ATAAATGAGAATGATTTTAATTAAT
A1S_2123	GATAATAAGAATTATTTTTATTTGT
A1S_2278	TTATTTGATAATGATTTTCATTTAT
A1S_2372	GTTATTGATAATAATAATCATTTGC
A1S_2382	GCAACTGGTAATCATTTTCATTTGT
A1S_2391	GTAATTGTAAATGATTATCATTTAT
A1S_2392	GTAAATAATAATCATTATTAATTGT
A1S_2567	TTACTTGAGAATGATTCTTGATAAC
A1S_2581	TTAAATGAGAATCATTTTCATTTAT
A1S_2582	TTAAATGAGAATCATTTTCATTTAT
A1S_2667	TTTTTTGAGAATTATTATTGATTAT
A1S_3174	ATTATTGATAATTATTATCGTTTGT
A1S_3324	GATAATGAGAATTATTTTAATTTAT
A1S_3339	TTAAATGATTATAATTATCATTTAT

In a previous study, the upstream regions of *FUR *genes from different *A. baumannii *isolates were aligned to obtain a motif representing the FUR binding site [26]. Some differences exist between this sequence and the FUR box sequence shown in Figure 5, most notably, the previously described motif lacked the typical FUR box palindomic structure. FUR is known to auto-regulate its own expression, however, as seen in the current study, up-regulation of *FUR *under iron limiting conditions is at lower levels than that of other FUR regulated genes, e.g., siderophore biosynthesis genes, a phenomenon that could reflect a lower binding affinity of the FUR protein for the *FUR *promoter region [35-38]. Therefore, the FUR binding sequences found upstream of the *FUR *gene may have predicted a less than optimal *A. baumannii *FUR box consensus motif, which does not show the typical palindromic structure. Moreover, FUR motifs of different bacterial genera show a high level of homology, whereas the previously described *A. baumannii *FUR motif is more distant.

A MAST search (parameters; E < 100, p < 10e^-4^) using the optimized *A. baumannii *FUR motif showed 81 hits to the *A. baumannii *ATCC 17978 genome (Additional file 2). Over 80% of the genes with a well conserved FUR box upstream (p < 10e^-5^, n = 41) showed more than 2-fold up-regulation. Furthermore, of the 95 genes up-regulated more than 4-fold under iron limiting conditions, 75 were preceded by putative FUR binding sites. These studies highlight a significant correlation between the level of conservation of a putative FUR binding site and the level of up-regulation under iron limiting conditions.

Extracytoplasmic function (ECF) transcription factors are sigma-70 family proteins that are responsive to environmental changes such as iron starvation [39]. These proteins play an important role in regulating iron-uptake mechanisms in several bacterial genera. For example, one of the best characterized ECF sigma factors, PvdS, controls the genes required for biosynthesis and transport of the siderophore pyoverdine in *P. aeruginosa *[40]. Expression of *pvdS *and various other sigma-70 factors in *Pseudomonas *is regulated by FUR [40]. However, in this study no predicted sigma-70 factors were identified in the list of genes containing a putative *A. baumannii *FUR binding site (Additional file 2). Moreover, no significant differentially expressed sigma-70 factors were found under the iron limited conditions, suggesting that in *Acinetobacter*, these proteins do not function in iron-uptake regulation. Another regulatory mechanism involved in iron homeostasis includes small RNA molecules, such as *ryhB *from *E. coli *or *prrF *from *P. aeruginosa*. However, sequences homologous to either of these small RNAs were not found in the *A. baumannii *ATCC 17978 genome. Nonetheless, a role for small RNAs in iron homeostasis can not be ruled out, since the *A. baumannii *ATCC 17978 genome contains a gene encoding the RNA chaperone Hfq (A1S_3785), which is required for the functionality of numerous small RNAs involved in iron homeostasis in various Gram-negative bacteria [41,42]. The results of this study suggest that FUR is the primary regulator of iron uptake in *A. baumannii*.

### Transcriptional profiling of the siderophore mediated iron acquisition mechanisms

**Figure 6:**
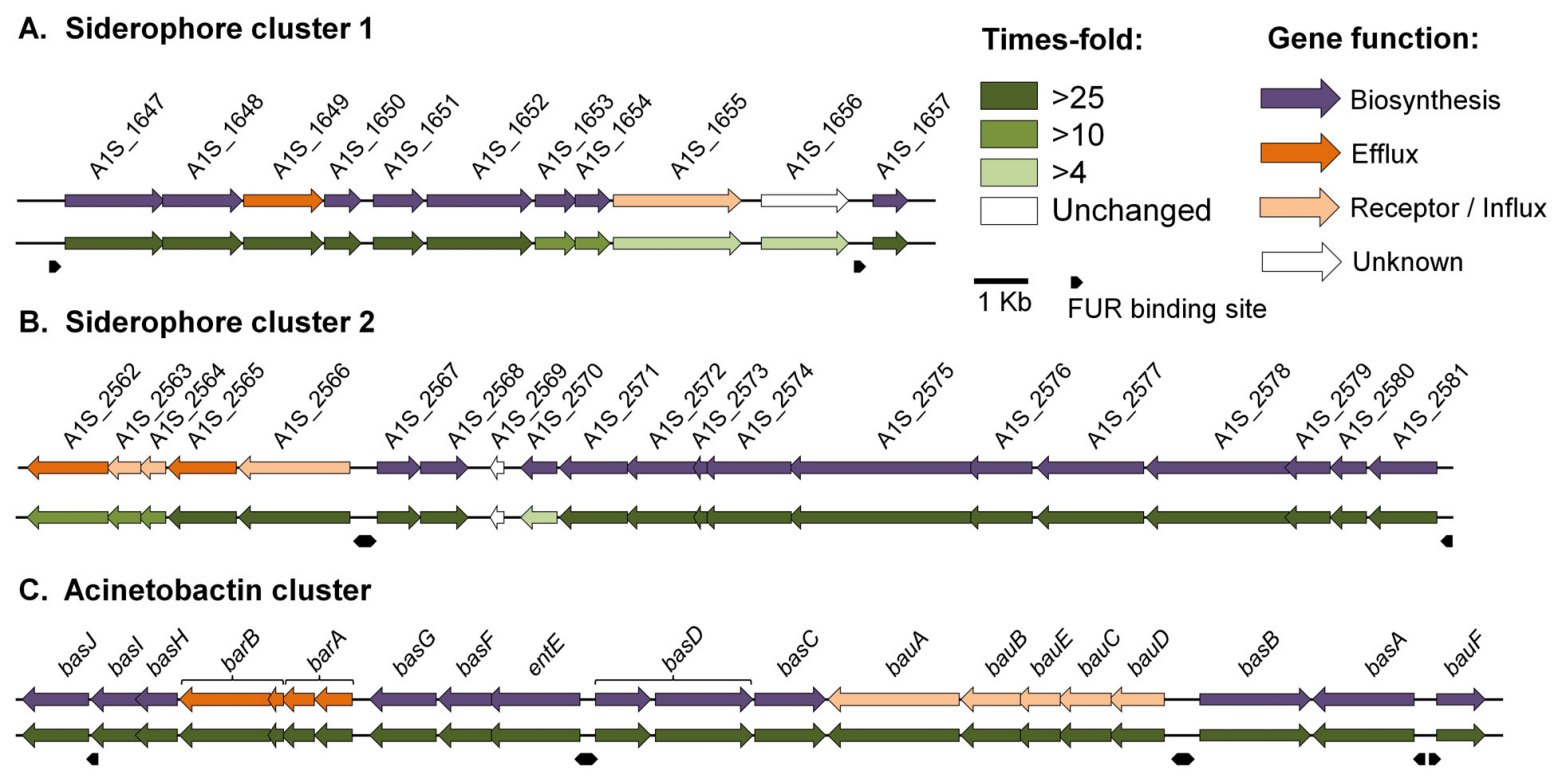
**Transcriptional profiling of three siderophore gene clusters identified in *A. baumannii *ATCC 17978**. Transcriptional alteration of the three siderophore gene clusters to low iron conditions are shown, (A) siderophore gene cluster 1 (A1S_1647-1657), (B) siderophore cluster 2 (A1S_2562-2581) and (C) the acinetobactin gene cluster (A1S_2372-2392). The top arrows show predicted gene function; siderophore biosynthesis in purple, receptors and uptake mechanisms in light orange, efflux pumps in orange and genes of unknown function in white. The relative transcriptional differences between *A. baumannii *grown under iron replete and iron limiting conditions are depicted in the bottom set of arrows according to the green color scale bar, all values are in times-fold difference. Genes depicted in white were not differentially expressed and those in dark green were overexpressed more than 25-fold. No significant down-regulation was observed within the siderophore gene clusters. Putative FUR boxes are shown as black arrows.

Genes involved in the biosynthesis, efflux and uptake of a siderophore are often clustered within bacterial genomes. To date, three putative siderophore gene clusters have been identified in *A. baumannii *[21-23], of which two can be found in strain ATCC 17978. A significant finding from the microarray results was the detection of the novel putative *A. baumannii *siderophore gene cluster (Figure 2; Siderophore cluster 1: A1S_1647 - A1S_1657). Several genes within siderophore cluster 1 were more than 100-fold overproduced under iron limitation, highlighting their potential importance in iron uptake (Figures 2 and 6a). Siderophore cluster 1 contains eight genes with a putative function in siderophore biosynthesis, A1S_1647, A1S_1648, A1S_1650-1654 and A1S_1657. Siderophore extrusion is most likely facilitated by an MFS efflux pump (A1S_1649). A receptor (A1S_1655) and PepSY-associated transmembrane helix family protein (A1S_1656) are likely to be involved in recognition and reduction of ferric siderophores, respectively. FUR boxes for transcriptional regulation of the unidirectional, operon-like gene cluster could be identified upstream of A1S_1647 and A1S_1657 (Figure 6a).

Siderophore gene cluster 2 (A1S_2562-2581) [23] showed similarly high levels of overexpression as cluster 1 (Figure 6b). This cluster contains 15 genes involved in siderophore biosynthesis (A1S_2567-2581), three genes involved in recognition and uptake of the ferric siderophore (A1S_2563, A1S_2564 and A1S_2566) and two genes encoding putative efflux pumps. One efflux pump gene, A1S_2565, encodes a putative MFS efflux pump. As mentioned previously, members of this family have been identified in various siderophore gene clusters in other bacteria and have proven to play a role in the efflux of enterobactin [14]. The second efflux pump, A1S_2562 is a member of the multidrug and toxic compound extrusion (MATE) family [43]. To our knowledge this is the first report of a bacterial MATE pump having a putative role in siderophore efflux. Similar to siderophore cluster 1, FUR appears to be the main transcriptional regulator, since binding sites could be identified upstream of A1S_2566, A1S_2567 and A1S_2581 (Figure 6b).

The most extensively characterized *Acinetobacter *siderophore gene cluster is that responsible for biosynthesis of acinetobactin [22]. Acinetobactin is synthesized from a 2,3-dihydroxybenzoic acid, threonine and hydroxyhistamine, and contains catecholate and hydroxamate groups that provide a high affinity for iron [25]. The acinetobactin biosynthesis genes include *basA-D*, *basF- J *and *entE *(Figure 6c). Gene pair *barA *and *barB *encodes a siderophore efflux system of the ABC-superfamily, the products of *bauA-F *form a receptor for recognition of ferric acinetobactin and the products of *bauB-E *are involved in translocation of ferric acinetobactin (Figure 6c). All of these genes showed high levels of overexpression, ranging from 43-fold to 165-fold. The cluster contains putative FUR boxes upstream of *basJ*, *entE*/*basD*, *basA*/*bau *and *bauD*/*basD*. These same FUR binding sites have been experimentally identified using a FUR titration assay [22], validating the FUR analysis described here.

Interestingly, transcriptional up-regulation gradually decreased in all three siderophore clusters when distance from the FUR box increased, demonstrating the importance of FUR in regulating siderophore production at the level of gene transcription. This is the first time that a full transcriptional profile has been provided for the siderophore gene clusters in *A. baumannii *under iron limiting conditions. Most importantly, a novel putative siderophore gene cluster was identified.

Siderophore receptors expressed on the surface of the bacterial outer membrane play a crucial role in the recognition of iron-loaded siderophores and therefore iron uptake. These receptors are likely to recruit the TonB-ExbB-ExbD translocation system for transport of ferric siderophores from the extracellular space to the cytoplasm [16,44]. The *A. baumannii *ATCC 17978 genome contains 22 putative siderophore receptors. Analysis demonstrated that 15 of these are located downstream of a putative FUR box (Additional file 2) of which 11 were significantly up-regulated under iron limiting conditions.

Under iron limited conditions, high levels of overexpression were also determined for the *tonB-exbB-exbD *gene cluster (A1S_0452-0454) which contained a predicted FUR box. Previously, a second TonB-ExbB-ExbD energy transduction system in strain ATCC 17978 (A1S_1603-1605) was described [23]. The cluster restored enterobactin utilization in *E. coli exbBD *mutants, but not in *tonB *mutants. However, in our study, no significant transcriptional up-regulation of the genes within this cluster (A1S_1603-1613) was observed under iron limiting conditions. It is possible that the cluster is related to heme acquisition rather than siderophore mediated iron uptake, since genes related to hemophore utilization were located adjacent to this cluster. A heme receptor/reduction mechanism may not be required under the conditions tested in our study, since no hemophores are being synthesized by *A. baumannii *ATCC 17978 and no exogenous hemophores were present.

### Investigation of motility under iron limiting conditions

It is well established that pili play an important role in the pathogenicity of bacteria due to their roles in motility, adherence, invasion and resistance [45,46]. Grouping transcriptome results from this study by COG function showed that 18% of the genes related to motility were significantly down-regulated under iron limiting conditions (Figure 4). The down-regulated genes from this group are part of the chaperone-usher pili assembly systems (type I pili) and type IV pili that have been previously identified in *A. baumannii *[47]. Homologous features have been associated with biofilm formation and motility in various organisms, including *E. coli *and *P. aeruginosa *[48-50].

**Figure 7:**
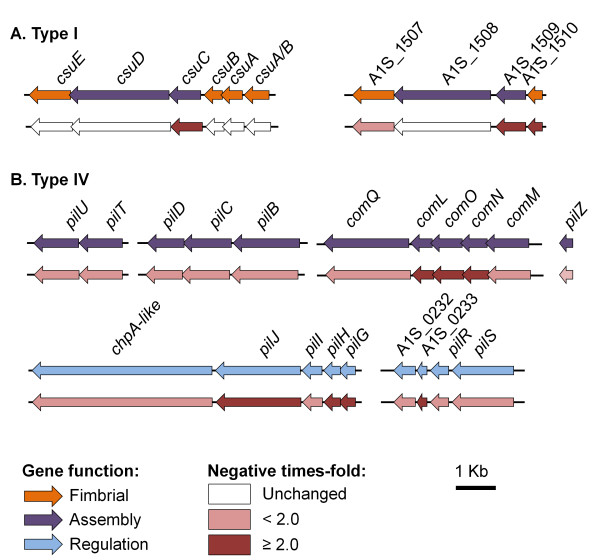
**Gene clusters with a putative role in motility**. Many genes in the motility COG were found down-regulated including genes of (A) type I and (B) type IV pili. The top set of arrows show predicted gene function; pilus proteins in orange, pilus assembly proteins in purple, and regulatory proteins in light blue. The relative transcriptional differences between *A. baumannii *grown under iron rich and iron limiting conditions is depicted in the bottom set of arrows, all values are in times-fold. Genes depicted in white were not differentially expressed, those shaded in light red were down-regulated less than 2-fold, whereas those in dark red were down-regulated 2-fold or more. No significant up-regulation was observed within gene clusters related to motility.

In *A. baumannii*, biofilm formation on abiotic surfaces has been linked to a type I pili encoded by *csuAB-E *[6]. The CsuA/B, CsuA, CsuB and CsuE proteins are predicted to form part of the type I pili rod [6,46]. CsuC forms a periplasmic chaperone protein that accelerates folding of the pilus rod subunits and CsuD is an outer membrane protein (OMP) responsible for assembly and extension of the pilus [6,46]. CsuD shares 40% and 45% amino acid sequence similarity with the OMP of two other type I pili mechanisms in strain ATCC 17978, A1S_1508 and A1S_2089, respectively. *csuC *and homolog A1S_1509 were both down-regulated under iron starvation, by 2.0-fold and 3.7-fold, respectively (Figure 7a). The *csuB *and *csuE *homologs within this second cluster, A1S_1507 and A1S_1510, respectively, were also significantly down-regulated under iron limitation. There were no genes down-regulated in the third type I pili cluster (A1S_2088-2091). It has been shown that transcription of the *csu *cluster in *A. baumannii *is controlled by the BfmRS two-component regulator [51]. However, no significant differential expression was observed for either gene encoding this system in this study. Biofilm assays were performed under iron replete and iron limited conditions in order to assess the impact of down-regulation of the *csu *cluster in the formation of these structures. However, no significant differences were observed between planktonic growth and biofilm formation under iron limiting or replete conditions (data not shown). A similar study with *P. aeruginosa *on the effect of iron limitation on biofilm formation showed that growth as a biofilm was impaired to greater extent than planktonic growth [52]. Interestingly, it was also shown that twitching motility was enhanced when iron was less readily available [52], which could correlate with the overexpression of the type IV pili observed in *Moraxella catarrhalis *under iron limitation [53]. These findings indicate that binding and adherence characteristics follow different regulatory pathways in *A. baumannii*.

**Figure 8:**
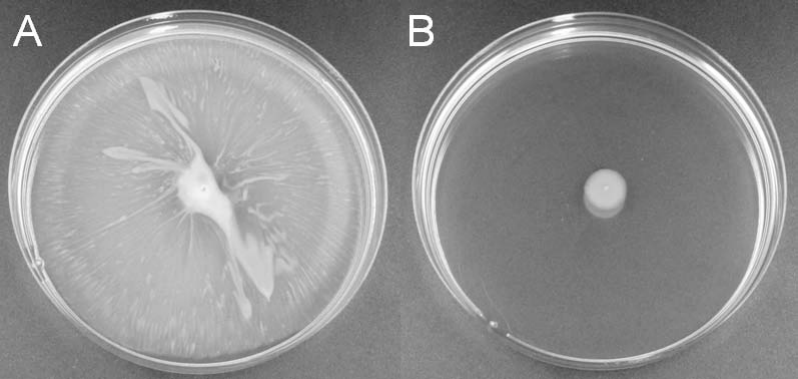
**Swarming of *A. baumannii *ATCC 17978**. *A. baumannii *colony material was spotted on Luria-Bertani medium containing 0.25% agar. Swarming motility (A) is visible as the channel-like growth around the dense white colony material. The absence of halo growth around the colony (B) indicates lack of swarming motility of *A. baumannii *when available iron is limited.

Various genes involved in the biosynthesis of type IV pili, including *pilB-D*, *pilT, pilU*, *comM-O*, *comL*, *comQ *and genes that play a role in chemosensory and regulation of this complex, *pilG-J*, *pilR*, *pilS *and the *chpA*-like, were down-regulated under iron limitation in strain ATCC 17978 (Figure 7b). In *P. aeruginosa*, type IV pili have proven to play a role in swarming motility, a form of migration over nutrient rich semi-solid surfaces [54,55]. Previous studies on the type IV secretion mechanism in *Acinetobacter *have been predominantly related to its function in DNA acquisition [56,57]. A swarming phenotype on Luria-Bertani medium containing a low percentage (0.25%) of agar was determined for *A. baumannii *strain ATCC 17978 (Eijkelkamp *et al.*, unpublished data). The effect of iron limitation on swarming motility was investigated by supplementation of DIP to the swarming medium. Strain ATCC 17978 was found to be incapable of migrating over the surface of the semi-solid medium when 200 μM DIP was supplemented in the medium, whereas non-migrational growth remained largely unchanged (Figure 8). Since bacterial motility is a high energy consuming process, the inability to migrate may be a stress response of *A. baumannii *ATCC 17978 to low iron levels.

### Comparative analysis of the iron acquisition mechanisms of sequenced *Acinetobacter *isolates

The iron uptake machinery encoded by different *A. baumannii *strains may differ, as variation in the composition of siderophore mediated iron uptake proteins in the outer-membrane has been shown in a study on different *Acinetobacter *strains [20]. Moreover, a siderophore gene cluster found in *A. baumannii *8399 (*om73 *- *entD*) could not be identified in any other sequenced *Acinetobacter *strain [21]. To explore this possibility in more detail comparative analyses of siderophore gene clusters were conducted using 10 fully sequenced *Acinetobacter *genomes, including *A. baumannii *strains ATCC 17978, ATCC 19606, AYE, AB0057, ACICU, 307-0294, D1279779, WM99c and SDF, and *A. baylyi *strain ADP1.

**Figure 9:**
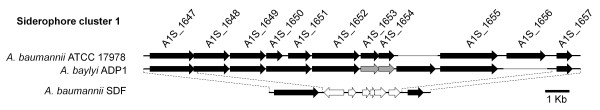
**Comparison of siderophore cluster 1 in sequenced *Acinetobacter *isolates**. The alignment of siderophore cluster 1 between strain ATCC 17978, ADP1 and SDF. Arrows indicate open reading frames, in black genes with high homology (> 50% identity) and in grey genes with low homology (< 50% identity). White genes represent a 3.5 Kb transposon insertion, which replaced the A1S_1648-1656 orthologs in strain SDF.

The novel putative siderophore cluster 1 (A1S_1647-1657) was found to be well conserved between strains ATCC 17978, ATCC 19606, AYE, AB0057, ACICU, 307-0294, D127 and WM99c (Table 2). Interestingly, the boundaries of this cluster, including genes orthologous to A1S_1647 and A1S_1657 that encode proteins with homology to the siderophore biosynthesis proteins IucA/IucC and acetyltransferase, respectively, were identified in strain SDF but the intervening genes appear to have been replaced by a 3.5 kb transposon encoding a transposase of the IS*5 *family (Figure 9). Well over 100 copies of this insertion sequence are found throughout the SDF genome and, along with other insertion sequence elements, are known to have played a major role in genome reduction in this environmental *A. baumannii *strain [47]. No other putative siderophore biosynthesis gene clusters were identified in strain SDF. Therefore, as a result of this insertion, this strain does not appear to encode any siderophore mediated iron acquisition mechanisms. This may be one reason that this strain has a higher requirement for soluble iron than other *A. baumannii *strains, as outlined below. Cluster 1 could also be found in *A. baylyi *APD1, however, it lacked a putative membrane protein (A1S_1656) and acetyltransferase (A1S_1657). Instead, the ADP1 cluster contained a putative acetyltransferase (ACIAD2117) inserted between orthologs of A1S_1654 and A1S_1655 (Figure 9). The putative membrane protein A1S_1657 is most likely involved in recognition of the chelated siderophore, however, this role could be fulfilled by other ferric siderophore receptors, encoded distally in the genome. Whereas, cluster 1 is in the same genomic position in all *A. baumannii *strains, in *A. baylyi *ADP1 it appears to have been subjected to genomic rearrangement as it is located in a different position.

**Table 2:** Genomic comparison of siderophore gene clusters in sequenced *Acinetobacter *isolates

		Cluster 1 A1S_1647-1657	Cluster 2 A1S_2562-2581	Acinetobactin A1S_2372-2392	Cluster 4 *om73*-*entD*	Cluster 5 ABAYE1888 and ABAYE1889
*A. baumannii*	ATCC 17978	+	+	+	-	-
	ATCC 19606	+	-	+	-	+
	AYE	+	-	+	-	+
	AB0057	+	-	+	-	+
	ACICU	+	-	+	-	+
	307-0294	+	-	+	-	+
	D1279779	+	-	+	-	+
	WM99c	+	-	+	-	+
	SDF	-	-	-	-	-
	8399	nd	nd	nd	+	nd

*A. baylyi*	ADP1	+	+	-	-	-

nd = not determined

Of the 10 *Acinetobacter *strains surveyed, siderophore cluster 2 was only detected in ATCC 17978 and ADP1. Additionally, no positive hits for A1S_2562, a gene within this siderophore cluster, were identified in 59 clinical *Acinetobacter *isolates from widespread locations in Australia using PCR screening (data not shown). Therefore, this cluster appears to be relatively rare across the *Acinetobacter *genus. The average homology between the ATCC 17978 and ADP1 siderophore cluster 2 genes was 75%, which is high compared to orthologous genes elsewhere within these two genomes, e.g. 53% within siderophore cluster 1. Transposases were found at the termini of siderophore cluster 2 in both ATCC 17878 and ADP1, suggesting that this gene cluster may have been horizontally acquired. Nonetheless, these transposases are distinct and have inserted into distinct genomic positions in the two strains, suggesting that siderophore cluster 2 was incorporated into the two genomes in separate transfer events. No other hits were obtained in a BLASTn search of the GenBank database with cluster 2, therefore, the origin of this cluster remains unknown.

A high level of conservation was observed for the acinetobactin gene cluster among most *A. baumannii *isolates (Table 2). However, this cluster was not seen in the SDF and ADP1 strains. A fifth cluster was identified by BLASTp searches in several sequenced *A. baumannii *strains (considering the *A. baumannii *8399 siderophore cluster as the fourth *Acinetobacter *siderophore gene cluster) (Table 2). The genes are represented in strain AYE by ABAYE1888 and ABAYE1889. A putative FUR box could be identified upstream of ABAYE1889 and high expression levels were observed under iron limiting conditions using qRT-PCR analysis (data not shown). The two genes encode proteins that were found to be homologous to an isochorismatase and a 2,3-dihydro-2,3-hydroxybenzoate dehydrogenase. The product synthesized by these two enzymes in this cluster, 2,3-dihydroxybenzoate, is an iron binding compound, but also a precursor component for more complex siderophores, like acinetobactin. This cluster is well conserved between strains AYE, AB0057, ACICU, 307-0294, ATCC 19606, D1279779 and WM99c.

The minimum inhibitory concentration (MIC) of DIP was determined for seven of the strains included in the genetic comparison (*A. baumannii *strains ATCC 17978, ATCC 19606, AYE, D1279779, WM99c and SDF, and *A. baylyi *strain ADP1). Growth of *A. baylyi *strain ADP1 and *A. baumannii *SDF was inhibited at lower levels compared to other strains. ATCC 17978 did not show higher MIC values for DIP compared to AYE, WM99c or D1279779, despite having three highly expressed siderophore gene clusters. Therefore, viability of *Acinetobacter *strains under varying iron concentrations does not appear to directly correlate with the presence or absence of siderophore gene clusters. Strains with higher OD_600 _values under iron replete conditions showed higher MIC levels for DIP.

### A second FUR-like transcription repressor

Various bacterial genomes contain multiple *FUR*-like genes. These *FUR *homologs often encode repressors with similar domains but with higher affinity for metals other than iron, such as zinc, manganese or nickel [58]. The *A. baumannii *AYE genome also encoded a second FUR-like regulator that has not yet been characterized, ABAYE1887. This gene was located adjacent to the putative siderophore genes described above (ABAYE1888 and ABAYE1889). Conserved domain (CD)-searches showed the highest homology with cd07153 (E = 1e^-10^), which can be found in FUR and other metalloregulatory proteins. A putative zinc uptake regulator (ZUR) can be found in a zinc-uptake gene cluster in strain AYE (ABAYE3726). Pairwise alignment of ABAYE1887 and *FUR *(ABAYE2920), and ABAYE1887 and *ZUR*, indicated higher homology for ABAYE1887 to *FUR *than to *ZUR*, showing 46% and 33% similarity, respectively. Little is known about auto-regulation of *FUR *homologs. In the case of ABAYE1887, a FUR box with a low p-value can be found less than 200 bp upstream of the start codon using the ATCC 17978 optimized FUR motif. Moreover, qRT-PCR analysis demonstrated that growth of *A. baumannii *AYE under iron limiting conditions resulted in approximately 164-fold up-regulation of ABAYE1887, whereas, *FUR *only showed 1.6-fold up-regulation. This second FUR-like gene can also be found in strains ATCC 19606, D1279779 and WM99c, and a truncated form in SDF. Further experimental work is required to determine if ABAYE1887 plays a role in transcription of genes related to iron acquisition.

## Conclusions

This study defined the global transcriptional response of *A. baumannii *to iron starvation. The up-regulation of three siderophore mediated iron acquisition systems was the predominant feature of this transcriptional response. The high level of overexpression of these systems under iron limitation, suggests that each is active in mediating iron uptake and therefore likely to be of importance to *A. baumannii *for survival in iron limited environments, such as human hosts. Several genes involved in other processes, such as respiration and electron transport were also significantly differentially expressed. Our data corroborate results from a recently published proteomic study of *A. baumannii *under iron rich and iron limiting conditions [59], such as up-regulation of the iron acquisition mechanisms and *fumC*, and the down-regulation of *fumA *and *ompW*. The abundance of putative FUR binding sites identified upstream of up-regulated genes highlighted a major role for this regulator in transcriptional up-regulation under iron limiting conditions. Various genes of the type IV pili were down-regulated under iron limiting conditions. This may in fact explain the inability of strain ATCC 17978 to migrate on semi-solid surfaces under low iron concentrations. Overall, the results indicated that *A. baumannii *is adaptable to an environment with limiting iron availability.

## Methods

### Bacterial strains and growth conditions

*Acinetobacter *strains were obtained from the following sources: ATCC 17978 [GenBank: NC_009085] and 19606 [GenBank: NZ_ACQB00000000] from the American Type Culture Collection (ATCC); SDF [GenBank: NC_010400] from the Collection de Souches de l'Unité des Rickettsies (CSUR), Marseille, France; AYE [GenBank: NC_010410] from Patrice Nordmann, Dept. Bacteriologie-Virologie, Hopital de Bicetre, Le-Kremlin-Bicetre, France; WM99c and ADP1 [GenBank: NC_005966] from Jon Iredell, Westmead Millennium Institute, Sydney, Australia; and D1279779 from The Menzies School of Health Research, Darwin, Australia. The ATCC 19606 genomic sequence was obtained from the NCBI REFSEQ database. *A. baumannii *strains D1279779 and WM99c were sequenced recently by 454 pyrosequencing (Paulsen *et al.*, unpublished data). These Whole Genome Shotgun projects have been deposited at DDBJ/EMBL/GenBank under the accession numbers AERY00000000 for WM99c and AERZ00000000 for D1279779. The versions described in this paper are the first versions, AERY01000000 and AERZ01000000, respectively. The scaffold sequences of these three strains were tiled to the ATCC 17978 genome using Mauve [60].

Iron limitation was achieved by growing *Acinetobacter *strains in Mueller-Hinton (MH) medium supplemented with 2,2'-dipyridyl (DIP). The growth curves were obtained by culturing *A. baumannii *ATCC 17978 with different concentrations of available iron in Mueller-Hinton (MH) medium; untreated, 100 μM DIP, 200 μM DIP and 300 μM DIP. In all following experiments, cultures in MH were supplemented with 200 μM DIP.

### Microarray development

An 8 × 15 K custom genomic microarray was developed for *A. baumannii *ATCC 17978 on the Agilent platform using the Agilent eArray package http://earray.chem.agilent.com/earray/. At least four 60 mer DNA oligonucleotides with an average GC % of 41.5, were incorporated into the design for each of the protein coding genes annotated in the ATCC 17978 genome sequence [61]. The array also included a set of intra array controls; 132 probes replicated at least 10 times in the design, and the Agilent control spots.

### RNA isolation

Cells cultured under iron replete, untreated MH, and iron limited conditions of MH with 200 μM DIP, were grown until they reached mid-log phase (OD_600 _= 0.7). The cells were pelleted and lyzed in 1 mL TRIzol^® ^reagent (Invitrogen, Australia). Following phase separation, RNA was extracted from the aqueous phase using the PureLink™ Micro-to-Midi Total RNA Purification kit (Invitrogen), incorporating an on-column DNAseI (Invitrogen) digestion, as per manufacturer's recommendations.

### cDNA synthesis and microarray hybridization

For the microarray analyses, the cDNA synthesis, labelling and hybridizations were conducted at the Ramaciotti Centre for Gene Function Analysis, University of NSW, Australia. Total RNA was reverse transcribed and labelled with either Cy3 or Cy5 using the Agilent Fairplay Microarray Labelling kit (Stratagene). Labelled cDNA samples were hybridized to a custom designed 8 × 15 K two colour gene expression microarray slide. The results reported are based on three biological and four technical repeats, including one dye-swap experiment. Statistical analysis was performed on log_2_-transformed signal ratios of the replicate spots using the SAM algorithms [27]. All results described were found to be significant using a false discovery rate of less than 5% unless otherwise indicated. All microarray data presented are in accordance with the Microarray Gene Expression Data Society's minimum information about microarray experiment recommendations [62]. Descriptions of the microarray experiments, quantification data and array design have been deposited into GEO http://www.ncbi.nlm.nih.gov/geo/ and can be accessed using the accession number GSE24921.

### Quantitative RT-PCR

Validation of the microarray results, preliminary experiments with *FUR *and transcription level measurements of *A. baumannii *strain AYE were performed using a two-step quantitive RT-PCR (qRT-PCR). RNA isolation was performed as described above. cDNA was synthesized using random hexamers and SuperscriptII (Invitrogen). Primers were designed to generate 100 - 150 bp amplicons and are listed in Table 3. qPCR was performed using Sybr Green mastermix (Invitrogen). Transcriptional differences were calculated using the ΔΔC_t _method [63].

**Table 3:** Oligonucleotides used in the study

Locus tag	Forward primer (5'-3')	Reverse primer (5'-3')
A1S_r01*^+^	CAGCTCGTGTCGTGAGATGT	CGTAAGGGCCATGATGACTT
A1S_2501^+^	CAACACTGGTAAATGGCGTG	ACAACGTTTTTCATTTCGCC
A1S_0395	TCATGCTCTTGTTCAGTGGC	GCATTGCCAATACCCCTAGA
A1S_3420	GCCTTGCTTTACTTGTTCCG	GCATCAGTAAATGGGCAGGT
A1S_2562	TTGCCATCAGTAGTGCAACC	TCCTGCAATCACAACACCAT
A1S_3371	CAGATCCAACTGTGGTGGTG	TCAGCATCGGTACGGTTACA
A1S_0895	GCGCAAAGCTGGACTTAAAG	CGGTAAACTGTCGCAAGTCC
A1S_2565	TGGCTCGATATTCAACGTCA	TAACAGCAAACCACCACCAA
A1S_0897	CCGCGAGCGACTAAGC	TGTCGCAGCCCATGAA
A1S_1647	GGACGCCATCGTCTCG	GCGTCCCGGCTTTGTA
A1S_1925	GGTGGCGCGCTATTTG	GTTGCGCCATTGGGTA
A1S_2080	GGTCGATGGCGTTCCA	CAGCCGCTTTCGTGGT
A1S_3195	GCGCTCAACCGCGTAA	TGCCGGATCGTCTTGC
A1S_1509	CCAAGGAAGGCGCTGT	TTGGGGAATGGCTTGC
ABAYE1887	CCCTTTTGATGATTTTACGG	CAAGGCTTAAGCGCGGTA
ABAYE1888	CCAGCGCATCACCACA	TCCGCTCGAACAACTCA
ABAYE1889	GGGGCGATTTCAAGTGC	TCGCGATCAGCCAACA

* Primer sequences obtained from Higgins *et al.*, 2004 [66].

^+ ^Oligonucluotides used as references.

### *A. baumannii *FUR binding site analysis

A scoring matrix was defined from the 48 experimentally determined *E. coli *FUR binding sites [34] using the Multiple Em for Motif Elicitation (MEME) tool. The *A. baumannii *ATCC 17978 genome was analysed with the resulting scoring matrix using the Motif Alignment and Search Tool (MAST). Putative FUR binding site sequences that are located within the 200 bp region upstream a start codon, with a p-value of less than 10e^-5 ^and of which the downstream gene showed more than 4-fold up-regulation were further investigated. Subsequent MEME and MAST analyses with the described criteria were performed until no new positive hits were obtained. The resulting 21 putative FUR binding site sequences were aligned using Weblogo 3.0 [64] to create the optimized *A. baumannii *FUR motif.

### Swarming motility assay

Swarming motility assays were performed at 37°C on Luria Bertani (LB) medium containing 0.25% agar. Positive swarmers showed a halo growth zone with channel-like structures.

### Static biofilm formation assay

The static biofilm formation assay was performed as described previously [65] with minor modifications. MH broth was inoculated with bacterial colony material and incubated overnight at 37°C. The cultures were subsequently diluted 1:100 in fresh MH broth in polystyrene microtiter trays and incubated ON at 37°C. Adherent cells were washed once with PBS, stained by incubation with 0.1% crystal violet for 30 min at 4°C, and washed 3X with PBS. Dye was released from the cells using ethanol:acetone (4:1) and shaking at 200 rpm for 30 min at RT. Absorbance was measured at 595 nm on a Fluostar Omega spectrometer (BMG Labtech, Offenburg, Germany). The biofilm data represent the average of at least 3 independent experiments of triplicate wells.

## Authors' contributions

BAE, KAH, ITP and MHB designed the research project. BAE and KAH carried out the experiments and BAE, KAH, ITP and MHB wrote the manuscript. All authors have read and approved the final manuscript.

## Supplementary Material

Additional file 1 **Significance analysis of the microarray results**. Significant differentially expressed genes in the microarray, determined by SAM [27]. Click here for file

Additional file 2 **ATCC 17978 FUR binding sites**. A list of putative FUR binding sites in the *A. baumannii *ATCC 17978 genome determined by MAST with E < 100. Click here for file
